# Patterns of HIV-1 Protein Interaction Identify Perturbed Host-Cellular Subsystems

**DOI:** 10.1371/journal.pcbi.1000863

**Published:** 2010-07-29

**Authors:** Jamie I. MacPherson, Jonathan E. Dickerson, John W. Pinney, David L. Robertson

**Affiliations:** 1 Faculty of Life Sciences, Michael Smith Building, University of Manchester, Manchester, United Kingdom; 2 Centre for Bioinformatics, Division of Molecular Biosciences, Imperial College London, London, United Kingdom; Imperial College London, United Kingdom

## Abstract

Human immunodeficiency virus type 1 (HIV-1) exploits a diverse array of host cell functions in order to replicate. This is mediated through a network of virus-host interactions. A variety of recent studies have catalogued this information. In particular the HIV-1, Human Protein Interaction Database (HHPID) has provided a unique depth of protein interaction detail. However, as a map of HIV-1 infection, the HHPID is problematic, as it contains curation error and redundancy; in addition, it is based on a heterogeneous set of experimental methods. Based on identifying shared patterns of HIV-host interaction, we have developed a novel methodology to delimit the core set of host-cellular functions and their associated perturbation from the HHPID. Initially, using biclustering, we identify 279 significant sets of host proteins that undergo the same types of interaction. The functional cohesiveness of these protein sets was validated using a human protein-protein interaction network, gene ontology annotation and sequence similarity. Next, using a distance measure, we group host protein sets and identify 37 distinct higher-level subsystems. We further demonstrate the biological significance of these subsystems by cross-referencing with global siRNA screens that have been used to detect host factors necessary for HIV-1 replication, and investigate the seemingly small intersect between these data sets. Our results highlight significant host-cell subsystems that are perturbed during the course of HIV-1 infection. Moreover, we characterise the patterns of interaction that contribute to these perturbations. Thus, our work disentangles the complex set of HIV-1-host protein interactions in the HHPID, reconciles these with siRNA screens and provides an accessible and interpretable map of infection.

## Author Summary

HIV-1 is responsible for millions of deaths every year by causing acquired immunodeficiency syndrome (AIDS). Therefore, research is ongoing in order to better understand and counter HIV-1 infection. Like any virus, HIV-1 must enter host cells and use cellular machinery to replicate. To do this, proteins of the virus interact with the proteins of the cell. Many studies have identified specific virus-host protein interactions and a database known as the HIV-1, Human Protein Interaction Database was created for reference and further study to understand HIV-1 infection. In this work, we use the HHPID to find significant patterns of HIV-host interaction in order to identify core processes that are active during infection and also to highlight host cellular subsystems that are affected by HIV-1. We discuss the importance of these subsystems and associated interactions—in particular, whether the host proteins are supported by other recent data sets that were designed to find host factors essential for HIV-1 replication. We highlight mechanisms from essential steps in the viral life cycle as well as perturbations of the host immune response. Our work provides an accessible insight into HIV-1 infection.

## Introduction

Acquired immunodeficiency syndrome (AIDS), caused by HIV-1, is responsible for millions of deaths every year. Therefore, research into HIV-1 biology is of critical importance and research efforts are significant and ongoing. In order to replicate, HIV-1, like all viruses, must use host-cellular machinery and induce production of viral genomic material, viral proteins and ultimately new virions. This hijack and control over host cell processes is mediated by HIV-1 proteins through a complex network of molecular events, including virus-host protein-protein interactions (PPIs) [1]. Therefore, by developing our knowledge of the virus-host interaction network, we can improve our current model of HIV-1 infection and host-cell perturbation and use this information to aid development of new antiviral treatments. One example of a successful antiviral treatment that has come from understanding HIV-host cell interaction is the drug maraviroc [2]. Maraviroc is an entry-inhibitor that binds the CCR5 co-receptor, inhibiting gp120:CD4:CCR5 complex formation and, thus, entry into the host cell. Targeting a host protein in this way demonstrates that the number of possible HIV-1 therapeutic drug targets is not limited to the small viral proteome and that understanding the virus-host interface can lead to the development of novel-acting therapeutic agents.

Our knowledge of HIV-1-host PPIs is extensive in relation to other pathogens [3]. A major source of HIV-1-host protein interaction data is the HIV-1, Human Protein Interaction Database (HHPID) [1], [4], [5]. This database holds over 5000 interactions involving over 1400 human proteins, curated from primary literature on small-scale protein interaction studies. In the HHPID, an impressive level of detail is recorded, including a short description of each interaction outcome, e.g., ‘phosphorylates’, ‘binds’, ‘activates’ etc. However, there are several problems associated with this data set:

- Interactions in the HHPID come from a large number of separate publications over a wide date range and are derived from a diverse array of experimental procedures, such that the quality of the data is varied and the proportion of false-positive interactions, though presumably minimised by the small-scale nature of the contributing works, is difficult to estimate.
- The manual curation step introduces a potential for inconsistency and some anomalies have been identified [6].
- The database contains a large amount of redundant data, where the same interaction has been reported more than once in two separate records. For example, in the HHPID there are 27 entries describing interaction between the HIV-1 Tat protein and the human CDK9 protein, including five that describe binding and five more that describe complexing, two describing activation and three describing stimulation, although from these data it is not clear whether more than one interaction actually occurs.
- A second level of redundancy exists due to downstream consequences of interactions. For example, the finding that HIV-1 gp120 interaction with CD4 alters the activity of transcriptional regulators and cytokine transcription [7] is present as nine entries in the HHPID, when this activity can be explained through a direct interaction at the cell surface, causing downstream effects in the T cell receptor signaling pathways. However, by simply taking direct interactions from this database to determine host cell perturbation, important regulatory effects may not be considered or alternatively, perhaps falsely extrapolated.

Due to these reasons, while the HHPID is a unique, detailed source of individual PPI interactions that represents a large proportion of the knowledge in the published literature, it does not immediately provide a logical and functional map of HIV-1-host interaction.

Recently, three high-throughput HIV-human protein interaction data sets have been published that are the result of individual genome-scale siRNA gene knockdown screens [8]–[10]. These studies each identify over 200 host-cellular factors that are necessary for HIV-1 replication, termed ‘HIV-dependency factors’ (HDFs) [8]. A thorough meta-analysis of HDFs has been performed by Bushman *et al.* [11]. Though the pairwise intersection of genes between the three sets of HDFs is statistically significant in all cases [11], the number of genes confirmed by more than one study is only 34 and just three genes are present in all sets. This seemingly small overlap is largely thought to be due to differences in experimental procedure, including cell-type, choice of time points analyzed and choice of filtering thresholds [1], [9]–[11]. Despite the apparent small overlap between HDF sets, Bushman *et al.* demonstrate that certain cellular subsystems are mutually identified, such as DNA repair and nuclear transport associated proteins. This indicates the validity of the screen results and the value that can be gained by combining these data to identify essential host-cellular functions required by HIV-1 for replication. In addition, their study shows that intersections between HHPID data and the HDF sets, while significant are quite small at 39 [8], 54 [9] and 39 [10] genes. However, while the work of Bushman *et al.* successfully consolidates information between HDF sets and validates these sets against the HHPID, the underlying differences between the HHPID and siRNA screen results have not been explored in detail. In particular, cellular subsystems prevalent in the HHPID, but not present among HDFs, have not been identified.

Our previous visualisations of the HIV-human PPI network show that there are noticeable clusters of host proteins that take part in multiple interactions with the same set of HIV-1 proteins [4], [12]. These groups possibly represent multiple interactions with biologically related proteins, e.g., from functional pathways or protein complexes. In addition, highly connected subnetworks of host proteins, where some proteins are involved in multiple HIV-human interactions, have also been identified using a combination of human-human PPI data and HIV-human interaction data [9], [11]. These subnetworks represent specific biological activities including the ubiquitin-proteasome pathway, transcription, nucleic acid binding and nuclear import; all thought to be important in facilitating the early stages of HIV-1 infection [9]. However, in all of these studies, different types of HIV-1-host interaction are not taken into consideration in the clustering method, despite the potential for interactions to be quite dissimilar. For example, subnetwork PPIs may include direct binding interactions, indirect regulatory interactions and those with opposing actions, e.g., inhibition and activation, such that no systematic outcome is identifiable.

In this work, we explicitly utilise host-virus interactions and interaction types, as provided in the HHPID, to identify significant patterns of viral perturbation of the host cell. This permits us to gain meaningful insights into HIV-1 infection. Specifically, using a biclustering approach, we define sets of host proteins that take part in a common set, or ‘profile’, of HIV-1 interactions. Using a distance method to cluster these units, we identify higher-level groupings. We show that these higher-level groups of proteins map to specific biological subsystems in the host cell. By considering patterns of interaction with host cell proteins, evidence within primary literature and by assessment of support from global siRNA screens, we are able to infer the biological importance of these subsystems in terms of HIV-1 replication, host cell perturbation and regulation of the immune response. Thus, our work extracts a coherent functional map of core HIV-1-host interactions from the HHPID and consolidates findings from the major HIV-1-host PPI data sets.

## Results/Discussion

### Patterns of HIV-1-host interaction

We retrieved 1434 human proteins and 3939 distinct HIV-1-human PPIs from the HHPID. In order to precisely reflect findings from HHPID source papers and to maximise our capability to discern patterns within the data, all 19 HIV-1 proteins were used in our analysis. Not surprisingly, types of HIV-human PPI are not uniformly distributed among HIV-1 proteins, due to the different molecular functions of these proteins. We found that 18 from 19 HIV-1 proteins (all except Pol) take part in one or more interaction type with a frequency greater than expected by random chance (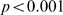). These over-represented interactions include 47 of the 68 interaction types given in the HHPID and 60 distinct interaction-type/HIV-1 protein combinations. To give some examples: (i) The HIV-1 protein retropepsin is a protease required in the HIV-1 life cycle to cleave viral polyproteins [13]. In addition, retropepsin cleaves proteins of the host cell [14]–[17], hence, retropepsin is responsible for all but one of 61 distinct ‘cleaves’ interactions. (ii) The HIV-1 accessory protein Nef can impact expression levels of multiple genes during the viral life cycle including proteases, cell-surface proteins, kinases, cyclins and transcription factors [18], [19]. Hence, Nef is responsible for a greater proportion of both upregulatory and downregulatory interactions than would be expected by random chance (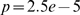). (iii) HIV-1 Tat is a transcriptional regulator that does not function alone [20], rather Tat works by recruiting other regulators [21], [22] and hence takes part in a greater proportion of interactions with type ‘recruits’ (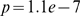) and ‘binds’ (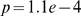). This over-representation analysis indicates that simple patterns of interaction (linking certain HIV-1 proteins to certain interaction types) are present in the HIV-1-host interaction network.

**Figure 1:**
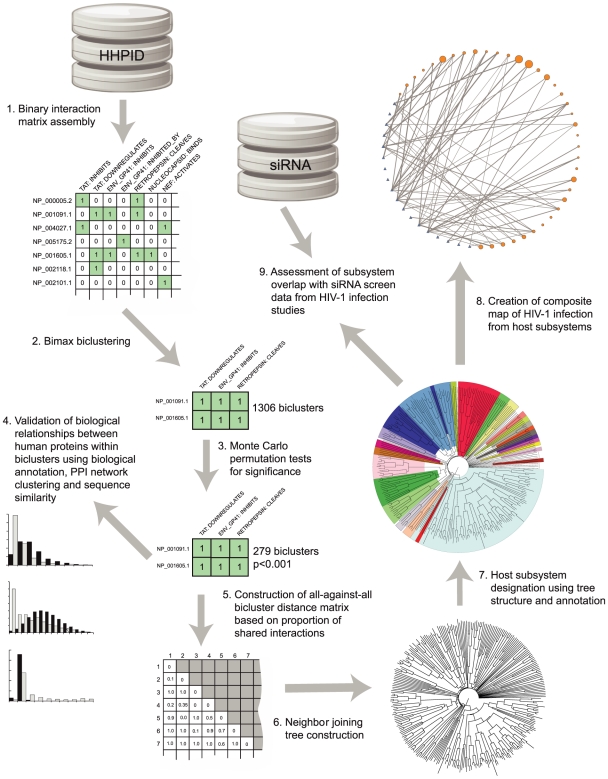
Summary of methodology. This diagram provides an outline of our method, steps are numbered according to the order in which they are discussed in the main text.

**Figure 2:**
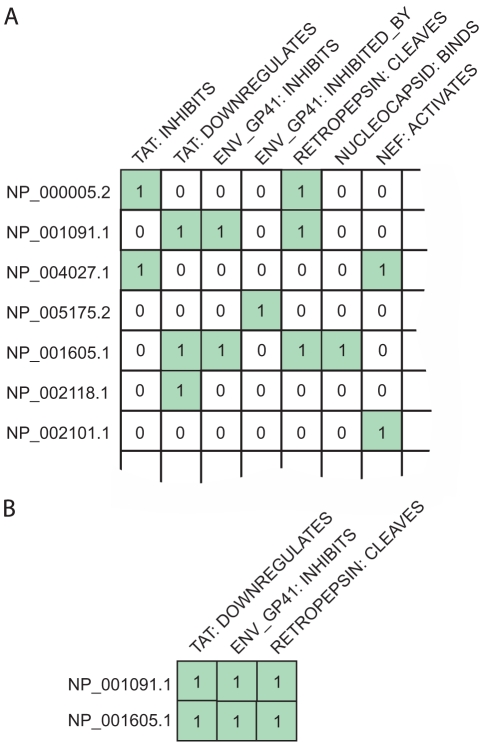
An example portion of the interactions matrix used in biclustering. (A) Shows an example portion of the interactions matrix. ‘1’ represents the presence of a given interaction, while ‘0’ the absence of that interaction, between a human protein interactant (shown left) and an HIV protein; the interaction having a given outcome (shown above). The entire matrix was biclustered to identify sets of host proteins that undergo the same set of HIV-1 interactions. (B) Shows an example bicluster that would be found in the portion of matrix given in (A).

To computationally identify more complex patterns of virus-host interaction, we investigated human proteins that take part in more than one distinct PPI with HIV-1 proteins. An outline of our method for analysis of HIV-1 interaction is given in figure 1. As a first step towards identifying key host functions known to be involved in HIV infection, we use biclustering to define groups of human proteins that share a common set (or ‘profile’) of HIV-1 interactions, in terms of HIV-1 protein interactant and interaction type (figure 2). The binary interaction matrix contained 1434 rows, 1292 columns and 3939 positive values, corresponding to human proteins, all types of HIV-1 interaction and all HIV-1-human PPIs, respectively. Biclustering of this matrix yielded 1306 biclusters that include a minimum of two human proteins, each with a minimum of two distinct HIV-1 interactions. We identified 279 from 1306 biclusters that were statistically significant (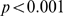) by Monte Carlo simulation. A table with details of all significant biclusters, including their constituent human proteins, HIV-1 proteins, interaction types and links to the HHPID are given in supplementary Table S1.

These biclusters define significant profiles of HIV-1 interaction and a corresponding set of human proteins, or termed differently, significant sets of human proteins that undergo similar perturbations during HIV-1 infection. Included in the significant biclusters were 246 human proteins, 18 proteins of HIV-1 (all except p6) and 1665 distinct HIV-1-human PPIs. According to the classes of bicluster, defined according to relationships between interactions, we found 122 independent, 137 mixed, 11 parental, 9 family and no sibling significant biclusters. Both independent and mixed biclusters, according to our interactions hierarchy (see supplementary Text S1), include a minimum of two unrelated types of PPI between every HIV-human protein pair. This indicates that our study of multiple interactions is informative and potentially valuable, as in 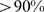 of cases, bicluster interaction profiles include two or more types of interaction that provide distinct, additional information regarding the perturbation of the human proteins.

We expected significant biclusters to be enriched for well-studied, high-confidence interactions, since they are likely to correspond to identifiable units of biological function and well established modules that have been investigated more thoroughly than smaller, insignificant biclusters or singleton interactions. This hypothesis was tested by counting publications that support the interactions, as given in the HHPID. Whilst we do not regard publication count to be an ideal measure, it is a reasonable and accessible estimate for confidence in a given PPI. We found that interactions within significant biclusters had a mean of 2.94 supporting publications, while other interactions with human proteins that could potentially be in biclusters (these take part in at least two distinct interactions with HIV-1 and are referred to as ‘potential bicluster proteins’ or PBPs) have a mean of 2.46 and interactions with all non-biclustered interactions had a mean of 2.29. Mann-Whitney U tests performed on the publication count distributions of biclustered interactions versus PBP interactions and biclustered interactions versus all non-biclustered interactions, demonstrated that the distributions were significantly different (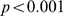, in both cases). While we do not suggest that interactions outside of these biclusters are false positives and that all interactions within these biclusters are of elevated importance, this finding does indicate that the overall patterns of interaction defined by significant biclusters that we discuss in this work, are likely to be biologically valid.

### HIV-1 interaction profiles define biologically cohesive sets of human proteins

To validate the biological significance of host protein sets and their associated interaction profiles (as defined by biclusters), we determined whether human proteins from within significant biclusters were more biologically similar to one another than expected by chance, assessed according to three measures: PPI network clustering to infer a greater then expected frequency of PPIs; semantic similarity in terms of shared Gene Ontology (GO) annotation [23] to infer shared biological roles; and sequence similarity to infer homologous relationships, as functional modules, such as protein complexes, are known to have a tendency to include paralogs [24]. These similarities were determined by comparing the host protein groupings to randomly selected sets sampled from 692 PBPs. Results for these measures are discussed below, followed by a summary of the three measures. In addition, detailed results, per significant bicluster, are given in supplementary Table S2.

### PPI network clustering

**Figure 3:**
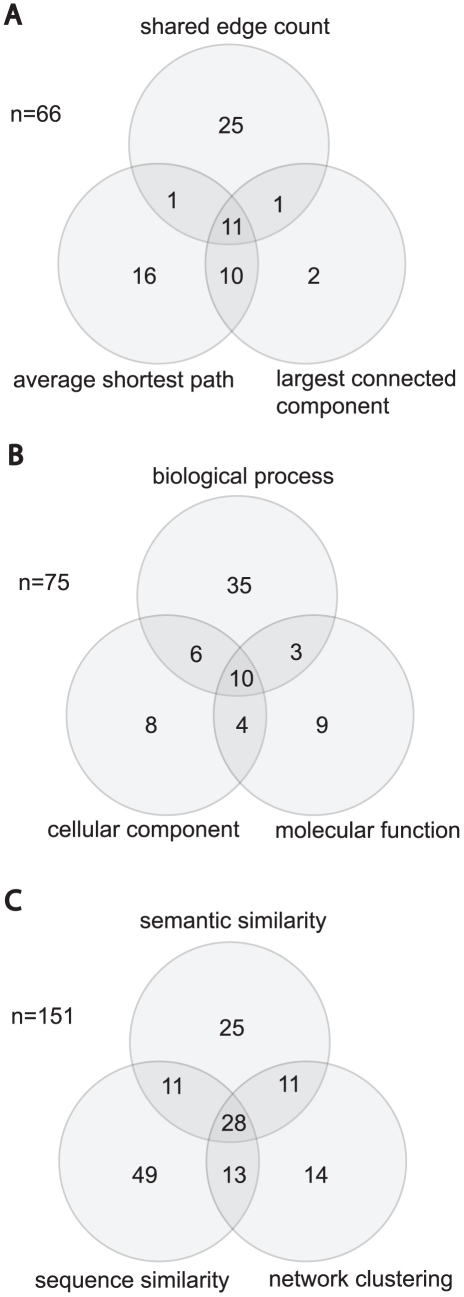
Venn diagrams showing biological cohesiveness among proteins within significant biclusters, using three measures. Counts refer to the number of biclusters that include human proteins that are significantly biologically related (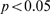) from a possible 279. (A) Displays three network clustering measures: shared edge count, average shortest path and largest connected component. (B) Displays semantic similarity in terms of the three GO ontologies. (C) Displays the overlap of all three measures of biological cohesiveness: semantic similarity, network clustering and sequence similarity.

Integrating human proteins from significant biclusters into a human PPI network, we identified 38 biclusters where the proteins share a greater number of interactions, 24 where the proteins form a bigger largest connected component (LCC) and 38 where the proteins have a smaller average shortest path length than would be expected by random chance (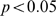). A total of 66 biclusters appear in the union of these three measures and figure 3A gives details of their intersection.

These results show that HIV-1 has a tendency to interact in similar patterns with host proteins that share interactions with one another, indicating the presence of multiple HIV-1 interactions with host protein complexes or other closely associated host network modules. There are several prominent examples of complexed proteins that constitute all of the host proteins defined by significant interaction patterns including: class II major histocompatibility complex (MHC), general transcription factor IIH (TFIIH), casein kinase II, adaptor-related protein complex 1, protein phosphatase 2A, N-methyl-D-aspartate receptor, microtubule subunits and RNA polymerase II (RNAP II). In some cases HIV-1 interaction patterns with these complexes become significant due to the number of subunits that undergo a set of interactions. For example, one significant combination of interactions acts upon nine subunits of the RNAP II complex, hence, these proteins have more shared edges than would be expected at random. In this case, the interactions are general, pertaining to the complex rather than being subunit specific, e.g., upregulation of RNAP II due to HIV-1 gp120 [25]. However, we also identify peptides from complexes that undergo subunit-specific interactions with the proteins of HIV-1. For example, one such bicluster involves HIV-1 Tat binding and regulation of specific polypeptides of TFIIH [1], [4], [5]. Yet, there are five other transcription-related host proteins within this interaction combination. In this case Tat interactions affect a functional module in the human PPI network (involving 18 interactions among the nine proteins, forming a single connected component) that corresponds to proteins of transcriptional regulation.

### Semantic similarity

**Figure 4:**
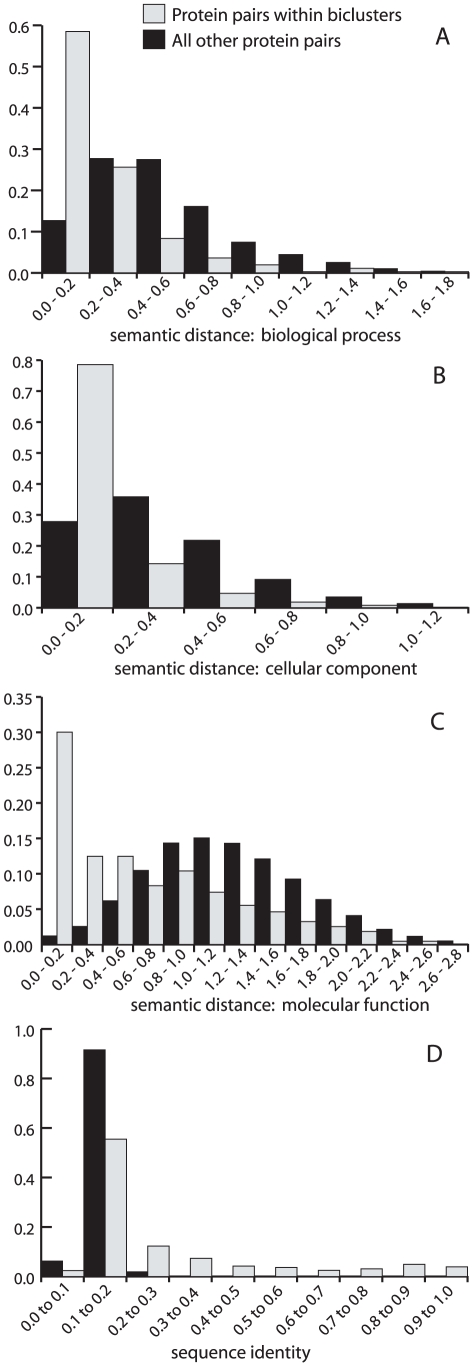
Comparison of protein pairs within significant biclusters to other protein pairs. Panels A, B and C show the semantic distance distributions for the three GO ontologies: biological process, cellular component and molecular function, respectively, for (i) human protein pairs from significant biclusters (shown in grey) and (ii) all other human protein pairs from PBPs (shown in black). Panel D shows the pairwise sequence similarity distributions for (i) and (ii). These charts show that human proteins from within significant biclusters are more similar in their GO annotation and sequence than other protein pairs (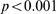 in a Mann-Whitney U test, in all cases).

Biclustered proteins are more similar in terms of their GO annotation than would be expected by random chance in all ontologies: molecular function, cellular component and biological function (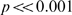). Semantic distance distributions for human protein pairs from within biclusters and all other PBP pairings, for each ontology are shown in figure 4, graphs A to C. We identified 75 significant biclusters that include human proteins that are significantly similar in their GO annotation, for at least one ontology, from a possible 204 significant biclusters that include two or more genes with GO annotation (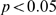). Details of the intersection between results for each ontology are given in figure 3B.

These results show that HIV-1 interacts in a similar pattern with proteins that have similar GO annotation. We are able to observe these similarities in all GO ontologies. For example, protein kinase C (PKC) isoforms that comprise all human proteins of one bicluster are annotated with the molecular function ‘protein kinase C activity’. Some cellular component GO terms refer directly to protein complexes. Certain biclusters involving complexes are therefore linked via common annotation, such as one that corresponds to RNAP II, annotated with GO term ‘RNA polymerase complex’. Interestingly, we are also able to observe HIV-1 interaction patterns that act upon specific biological processes including the immune response, protein kinase cascades, lipid modification, transcription, nuclear import and microtubule-based movement. The combinations of interaction that affect these processes can highlight the molecular methods through which HIV-1 infection perturbs cellular processes.

### Sequence similarity

Human protein pairs within significant biclusters are more similar in their protein sequence than would be expected by random chance (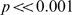). Distributions for sequence identity between human protein pairs from within biclusters compared to random pairings are shown in figure 4D. We identified 101 significant biclusters where the human proteins were more similar in their sequences than would be expected by random chance (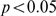). No biclusters were significantly less similar in their human protein sequences than would be expected.

We identify 58 biclusters for which a group of homologous proteins comprises more than half of the members of that cluster. We defined these homologous relationships by performing single linkage clustering on proteins, where proteins are linked if they share 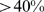 sequence identity. This cutoff was chosen as previous work has demonstrated that 40% sequence identity can accurately infer homology without the inclusion of an unacceptable proportion of false positives [26]. We found that significant biclusters with greater than expected sequence similarity among their host proteins (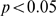) were also more likely to have at least one direct physical HIV-1 PPI (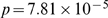) and the mean average proportion of direct HIV-1 PPIs among this group of biclusters was 25.5%, as opposed to 11.8% for all other significant biclusters.

These results show that paralogous groups of host proteins have a tendency to be subject to the same combinations of regulatory and physical HIV-1 interaction. Regulatory effects of HIV-1 interaction may be maintained across these groups, perhaps through stimulation of specific pathways. For example, isoforms of PKC, a kinase found to act in many signaling cascades [27], are the only host proteins among three particular significant biclusters. HIV-1 gp120 has been shown to upregulate multiple isozymes of PKC, possibly through classical signal transduction pathways [28], induced by binding to cell-surface receptors such as CCR5 [29]. However, the prominence of direct physical interactions among these homologous sets of proteins implies that there are conserved binding domains on members of closely related homologous groups, to which a HIV-1 protein can bind. For example, HIV-1 Vpr is designated in the HHPID to bind both importin-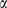 1 and 2 isoforms; as these proteins are 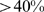 similar in a pairwise alignment, it seems likely that Vpr would bind a particular conserved domain of these proteins. However, various members of protein families can exert distinct phenotypic responses. In the case of PKC isoforms, cellular localisation and activation input can be controlled by the specific domain structure [27]. Different members of protein families may also exert distinct phenotypic responses due to their cellular background, caused by differential expression, but also by activating downstream targets to different quantitative levels, as shown for receptor tyrosine kinases [30], [31]. Therefore, to precisely determine HIV-1 perturbation, it remains important to distinguish what protein isoforms and family members are dysregulated and in what cell type this activity occurs.

### Summary for measures of biological cohesiveness

A summary of results from the three measures of biological relationship between proteins, in terms of the number of significant biclusters, is given in figure 3C. We find 151 from 279 biclusters are significant by one or more measure. Therefore, these measures are not mutually exclusive. In fact, in some cases, overlap may be due to a single biological phenomenon, e.g., homologous proteins that form a single complex are likely to be involved in the same biological process, in the same cellular compartment, possibly with the same molecular functions. For example, transcriptional regulators CREB binding protein (CBP), E1A binding protein (p300) and cyclin T1 are all found in one such bicluster whose interaction profile includes binding of these proteins to HIV-1 Tat and Vpr. CBP and p300 are 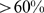 identical in local pairwise alignment, however, rather than binding Tat individually, they form a dimer (known as PCAF) [32]. Cyclin T1 shares only a low level of sequence similarity (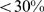 identity in local pairwise alignment) to the other two proteins. Therefore among these three host proteins there is a known PPI, a homologous relationship and all are transcriptional regulators involved with Tat mediated transactivation of the HIV-1 LTR [33] and hence have some common GO annotation. Furthermore, gene annotations including GO and PPIs may be attributed based on homology to genes with experimentally validated actions, for example, GO evidence code ‘ISA’, stands for ‘inferred from sequence alignment’ and is one of six codes describing computational assignment of annotation. Hence, the measures used here are linked. Some annotation is electronically inferred without any manual curation and as a result is error-prone [34]. In addition, false positive annotations can be propagated electronically [35], [36]. However, we chose not to select manual annotation alone as the potential reduction in false-positives is offset by an increase in false-negatives. For example, more than half GO annotations of human genes have the evidence code ‘IEA’ meaning ‘inferred from electronic annotation’ (see http://www.geneontology.org/GO.current.annotations.shtml).

We do not identify significant biological relationships among 128 biclusters. These biclusters include significantly fewer human proteins on average (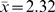) than the 151 biologically cohesive biclusters (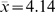) (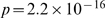, Mann-Whitney U test). Therefore, power to detect statistically significant biological relationships (despite their possible existence) among human proteins of these biclusters, is diminished, especially where annotation is lacking. For example, two subunits of the casein kinase II complex (alpha 1 and beta) are found in one such bicluster. At the time of performing this work neither of these subunits were GO annotated, they are not more than 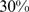 similar by local pairwise alignment and though they interact, this is insufficient to be called statistically significant. However, in some cases, no biological relationship can be discerned, even on inspection. Yet, of these 128 biclusters, 125 include fewer than three human proteins and none include more than four. This indicates that our combination of methods for detecting biologically cohesive human protein sets via biclustering, and detecting biological relationships among these biclusters, performs well in terms of quality, where the number of human proteins is four or greater.

### Host functions among HIV-1-host interaction combinations

Owing to the specific biclustering method that we used for defining significant profiles of HIV-1-host interaction, multiple biclusters arise from slight differences between protein sets that are essentially similar in their interaction profile. This is partly due to differently annotated interactions, interactions that are not maintained across a group of otherwise similarly interacting proteins, or even to missing interactions that have not been experimentally proven or are missing from the HHPID, i.e., false negatives. For example, in the case of two biclusters that include homologs of Akt (also known as protein kinase B), one pertains to homologs 1 and 2, the other to homologs 1, 2 and 3. These two biclusters occur because homologs 1,2 and 3 have been shown to share similar interactions with HIV-1 gp120 and Vpr. However, while homologs 1,2 and 3 are activated by Tat, only homologs 1 and 2 are shown to be upregulated by Tat in the HHPID [1], [4], [5]. Therefore, by combining biclusters according to shared information, we can form an overview of HIV-1 interactions with a given set of host proteins.

**Figure 5:**
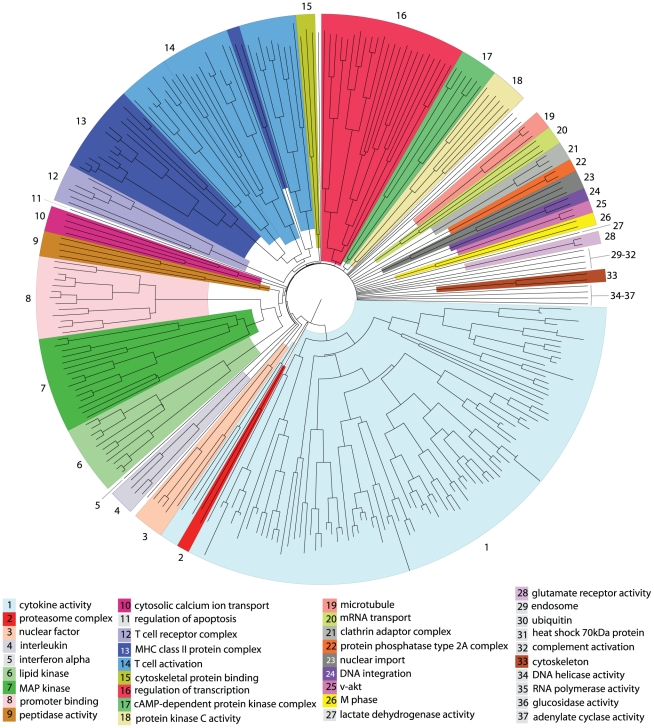
Tree showing the relationship between significant biclusters and higher-level host subsystem groupings. Individual biclusters are represented by terminal branches. Relationships are derived using a distance measure based on the proportion of shared interactions between significant biclusters and the tree was drawn using the neighbor joining method. The tree is divided into sections that show the higher-level host subsystems, largely derived using the tree structure. Subsystems of 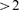 biclusters are colour coded (see key). Biclusters not labelled are those that have been placed in a biologically related group not adjacent on the tree.

Higher-level relationships between biclusters were identified using a distance measure based upon overlap between biclusters. Using the resulting pairwise distances a tree was constructed using the neighbor joining method [37] (see figure 5). This tree has been partitioned into sections, representing 37 biological subsystems within the host cell that are named according to over-represented GO terms, or after a specific protein (see materials and methods for more detail). In the tree representation we can observe subsystems that undergo a complex set of interactions during HIV-1 infection. These have a large number of terminal branches, representing many distinct but related HIV-host interaction combinations, where a single and clear pattern of interaction can not be simply defined, or does not exist, e.g., the *cytokine activity* subsystem. Conversely, the *v-akt* subsystem is relatively well defined including just two closely related HIV-host interaction combinations.

The identified subsystems and their associated patterns of interaction take place at a variety of levels within the host cell, including interactions at the cell surface and with specific biological components such as the proteasome. Cellular processes and pathways, including intracellular signaling cascades, apoptosis pathways and stimulation of the immune response, better describe other subsystems. In addition, some subsystems can be directly mapped to specific steps in the viral life cycle, including viral budding and transport of viral RNA across the nuclear membrane. Supplementary Table S3 gives details of each subsystem, including the number of biclusters, host and virus proteins. Supplementary Table S1 links individual biclusters and interactions to these subsystems.

Among these subsystems, there appears to be a central pathway of T cell signaling interactions that are perturbed by the proteins of HIV-1 at multiple levels in the cell. This pathway begins with inhibition of cell-surface receptor mediated signaling. For example, HIV-1 gp120 binding to CD4 prevents typical host-host cell-surface interactions, such as MHC-class II response to antigen binding [38], CD28-mediated co-signaling [39], and CD3-induced leukocyte-specific protein tyrosine kinase (Lck) and phospholipase C activation [40], . In addition, HIV-1 Nef downregulates CD4, CXCR4, CCR5, CD28, CD71, CD80, CD86 and MHC class I molecules via endocytosis [42]–[47].

We find continued perturbation of T cell signals at other cellular locations. For example, in the *MAP kinase* subsystem we find Lck, a component of TCR signaling and an activator of other cell signal transduction proteins including the ERK family of MAP kinases [48]–[51], is activated through gp120 binding to CD4 [52]–[54]. HIV-1 Nef also plays a role in the activation of the classical MAP kinase pathway via binding and activation of Lck [55], [56] and also Vav, causing downstream activation of JNK MAP kinases [57], [58]. Stimulation of these signaling cascades by proteins of HIV-1 influences a variety of cellular responses that include activation of transcription factors, for example [54], [59]. The *nuclear factor* subsystem includes nuclear factors of activated T cells (NFATs), transcriptional regulators that induce production of cytokines [60], [61]. We observe that NFATs are enhanced or activated at several levels within the host cell, by HIV-1 proteins Vpr, Tat, Nef and gp120, causing dysregulation of cytokine production [62]–[67]. Altered cytokine signals will then be received by cell-surface receptors, thus, completing a cycle of viral perturbation.

**Figure 6:**
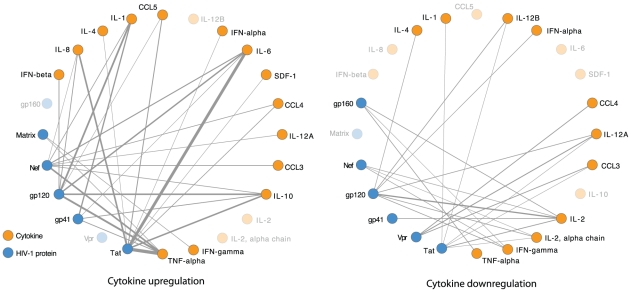
Cytokine regulation networks. These networks represent the pattern of cytokine regulation in the cytokine-activity host subsystem that were defined through identifying significant patterns of HIV-host interaction. Edges represent PPIs. Edge width is proportional to the number of PPIs being represented. For clarity, we only show PPIs that are reported more than once in the HHPID. These networks show that cytokine dysregulation due to HIV-1 infection is wide reaching and complex, affecting many host cytokines, both via upregulation (left) and downregulation (right).

To summarise the interactions between cytokines and proteins of HIV-1, we produced networks of both upregulation and downregulation, taking interactions from the *cytokine activity* subsystem, including interactions that are supported by more than one publication, as given in the HHPID (see figure 6). These networks illustrate the complexity of cytokine dysregulation by HIV-1. From 53 distinct HIV-protein, host-protein pairings in these networks, 30 pairs involve only cytokine upregulation, 12 pairs involve only cytokine downregulation and 11 pairs involve both cytokine upregulation and downregulation, in response to the HIV-1 protein interactions. Cytokine dysregulation is likely to have major pathogenic effects on the host system. For example, an increase in plasma levels of multiple cytokines during acute HIV-1 infection, coined an ‘early cytokine storm’, is associated with peak viral loads and immunopathological consequences [68].

In these network visualisations there are distinguishable patterns of cytokine regulation by HIV-1, such as, the largely stimulatory effects of gp120, Tat and Nef; upregulation of TNF-alpha and Interleukins 1 and 6; the repressive action of Vpr and gp160; and downregulation of interleukin 2 and interferon-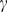. However, the overall picture of cytokine regulation during HIV-1 infection remains unclear. Future cytokine-wide studies of HIV-1 infected cells, ideally representing multiple different stages of infection and possibly even a variety of HIV-1 strains, coupled with an accurate model of cytokine action on the host system could improve our understanding of HIV-1 pathogenesis and potential intervention targets, particularly if key HIV-host interactions are identified.

**Figure 7:**
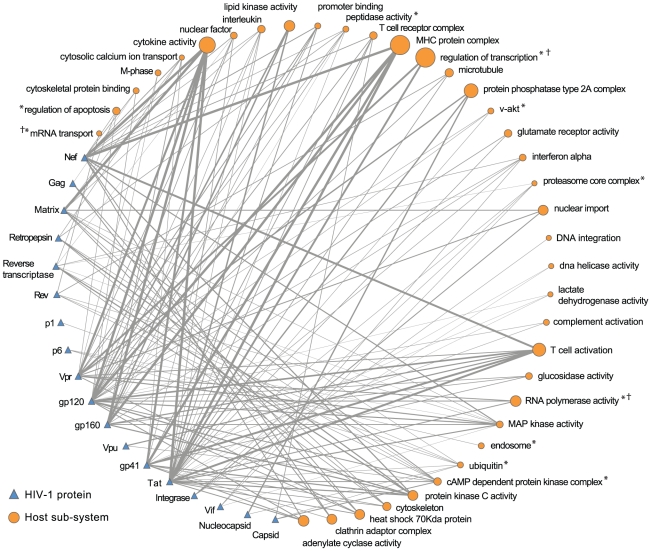
HIV-1-host interaction patterns, by HIV-1 protein. This network illustrates core patterns of HIV-host interaction. The human host is depicted as a series of cellular subsystems, represented by orange circular nodes, where the diameter of the node is proportional to the number of host proteins within that subsystem. HIV-1 is depicted by the viral proteome (blue triangles). Interactions between HIV-1 proteins and host subsystems are represented by edges, where the edge width is proportional to the number of interactions. For clarity, only those interactions that are shared by over half of the host proteins in a subsystem are shown. *Indicates a host subsystem whose subsystem annotation corresponds to a statistically significant group among HDFs (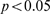). 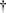 Indicates a statistically significant intersection between the subsystem proteins and HDF set (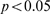).

**Figure 8:**
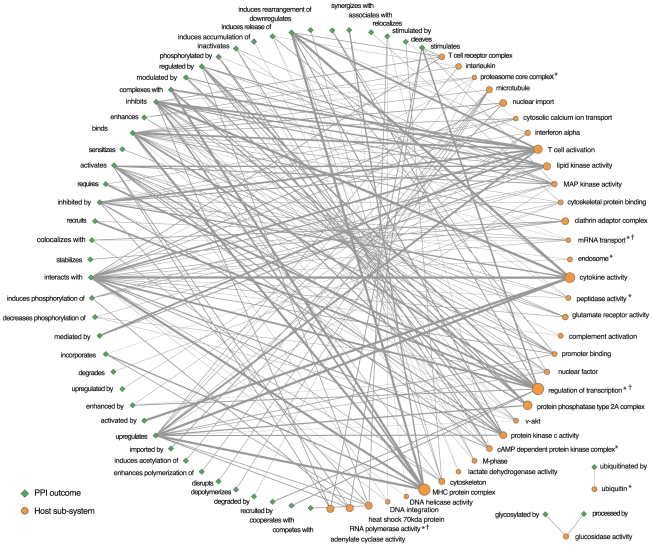
HIV-1-host interaction patterns, by interaction type. This network illustrates core patterns of HIV-host interaction. The human host is depicted as a series of cellular subsystems, represented by orange circular nodes, where the diameter of the node is proportional to the number of host proteins within that subsystem. The action that HIV-1 has on these subsystems is depicted by a series of interaction outcomes (blue diamonds). Interactions between HIV-1 and host subsystems are represented by edges where the edge width is proportional to the number of interactions. The directionality of the interaction is implicit in the description of the interaction outcome. For example, the edge linking the MHC protein complex node and the ‘upregulates’ node represents ‘HIV-1 upregulates the MHC protein complex’, whereas the edge linking the cytokine activity node and the ‘activated by’ node represents ‘HIV-1 is activated by cytokine activity’. For clarity, only those interactions that are shared by over half of the host proteins in a subsystem are shown. *Indicates a host subsystem whose subsystem annotation corresponds to a statistically significant group among HDFs (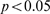). 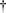 Indicates a statistically significant intersection between the subsystem proteins and HDF set (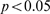).

To present distilled views of the HHPID and provide an interpretable network of HIV-1-host interaction, two HIV-1-host PPI networks were constructed. Both networks include 37 nodes that represent the characterised subsystems. The first network, shown in figure 7, has 18 nodes that represent the proteins of HIV-1. The second network, shown in figure 8 has 49 nodes that represent interaction types. The edges in these networks represent HIV-1-host interactions that contribute to significant biclusters, the width of each edge is proportional to the number of distinct interactions that are represented. Due to the condensed host functions and filtering out of patterns of interaction that are not statistically significant we can observe recognisable patterns of interaction in these networks. For example, (i) the relationship between HIV-1 Tat and regulators of transcription that are stimulated, activated and recruited by HIV-1 in the process of viral transcription. (ii) The multiple sources of perturbation of T cell activation from HIV-1 Nef, Tat and the envelope proteins. And, (iii) the large number of regulatory interactions between proteins of HIV-1 and host cytokines.

### Support for host subsystem functions among global siRNA data sets

To assess support for the 37 host subsystems from HDFs identified by global siRNA screens [8]–[10], we defined subsystem annotations that consist either of defining over represented GO terms or a regular expression that encapsulates a common protein name. Subsystem annotations are given in supplementary Table S3. We found that 10 from 37 subsystem annotations also define statistically over-represented groups among either all HDFs combined or a single HDF study (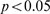). We find that 21 from 37 subsystems include at least one protein that is also present among HDFs and only in three cases is the intersection statistically significant (see supplementary Table S3 for more details).

### Cellular subsystems supported by HDF sets

The 10 subsystems that are supported by HDFs are: *proteasome core complex*, *regulation of apoptosis*, *mRNA transport*, *endosome*, *RNA polymerase activity*, *peptidase activity*, *regulation of transcription*, *ubiquitin*, *cAMP-dependent protein kinase complex* and *v-akt*.

Subunits of the proteasome core complex are present among two of the three siRNA screens [8], [9]. A meta-analysis of these HDF sets that incorporates data from the HHPID, showed that the proteasome is an important cellular component for HIV-1 replication [11]. The role of the proteasome in HIV-1 replication remains unclear. However, the interactions that we highlight between HIV-1 Tat and the beta-8 and beta-10 subunits may be important for determining proteasome composition, towards formation of the immunoproteasome, a change that may cause increased presentation of subdominant epitopes [69], [70].

Apoptosis is widely accepted as a mechanism for T cell depletion in HIV-1 infected individuals [71]. By reviewing relevant literature, we find that several subsystems may have a role in controlling apoptosis including: *regulation of apoptosis*, *glutamate receptor activity*, *v-akt*, *lactate dehydrogenase activity* and *peptidase activity*. HDFs found by one siRNA screen [10] are enriched for regulators of apoptosis. However, in our results, we only identify one HDF, Cytochrome C that is GO annotated as a regulator of apoptosis in addition to Akt and components of the glutamate receptor. We speculate that prevention, rather than induction of apoptosis, is an essential part of HIV-1 infection, in order to maintain a viral reservoir in the host [72]. In this case, HDFs may not include the pro-apoptotic host proteins that we observe in these interaction patterns. In addition, proteins such as Akt and Cytochrome C have roles outside of apoptosis [73], [74]. Therefore, the necessity for such proteins in HIV-1 replication is not necessarily apoptosis related. However, we identify subsystems from the HHPID that can be linked to positive regulation apoptosis, such as *regulation of apoptosis* that includes the activation of pro-apoptotic caspases by multiple HIV-1 proteins. The intensity of research to elucidate key interactions responsible for T cell loss, via apoptosis, in HIV-1 infected individuals is demonstrated by the prominence of pro-apoptotic HIV-host interactions in our results. However, we suggest that a greater range of interactions between proteins of HIV-1 and host regulators of apoptosis need to be investigated, particularly involving those host factors that are present among HDFs but not identified in our results.

Interactions in the *mRNA transport* subsystem all involve HIV-1 Rev. One of the roles of Rev is to facilitate export of HIV-1 RNA from the nucleus to the cytoplasm. A nuclear export signal present in the Rev protein binds to exportin 1, while an argenine-rich domain (ARD) in Rev binds to a Rev-response-element (RRE) present in viral RNA. To undergo nuclear export, an exportin-Rev-RNA complex docks at a nuclear pore complex (NPC), an interaction mediated by nucleoporins [75]. In the *mRNA transport* subsystem we find interactions that are specific to this process including but not limited to: binding [76] and recruitment [77] of exportin 1 by Rev; and direct interactions between Rev and two nucleoporin proteins [78], including Rev mediated recruitment of these host factors to the nucleus [79]. We find that there are a statistically significant proportion of host factors involved in mRNA transport in two of the three global siRNA studies (references [8], [9]). Furthermore, all host genes that make up this subsystem (a total of five) are found among HDFs (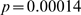). Down-modulation of these host factors, either in small-scale experiments or by global siRNA screen, apparently inhibits the interactions described in this subsystem, thereby preventing Rev-mediated RNA nuclear export and successful viral replication.

We observe two other subsystems that appear to have a role in transport of HIV-1 material into the nucleus, *nuclear import* and *heat shock protein 70kDa*. Briefly, the *nuclear-import* subsystem involves a variety of interactions with members of the karyopherin family and heat shock 70kDa chaperone protein (Hsp70). Karyopherins bind sequence motifs called nuclear localisation signals (NLS) of proteins, causing the protein to be directed into the nucleus [80]. We observe that HIV-1 Integrase, Matrix, Tat and Rev proteins are bound or imported into the nucleus by members of the karyopherin family. In the case of Integrase and Matrix, these interactions may relate to karyopherin mediated nuclear import of HIV-1 preintegration complexes (viral ds-RNA and associated proteins known as PICs) [81], a mechanism that may also involve HIV-1 Vpr. Several isoforms of the heat shock 70kDa chaperone protein (Hsp70) promote PIC import, possibly by stimulating interaction between PIC complexes and karyopherin [82]. These two nuclear import subsystems include support from siRNA screens. One of the three studies identified karyopherin-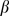 [9] and two studies [8], [9] identified transportin 3 (TNPO3), a less definitively characterised member of the importin-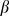/karyopherin-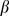 superfamily, as HDFs. More recently, TNPO3 has been reconfirmed by yeast-two-hybrid pull-down as a binding partner of Integrase, to be an early-stage HDF in the viral life cycle by siRNA screen and a clear promoter of HIV-1 PIC import [83], though subsequent work has shown that HIV-1 requirement for TNPO3 maps to interaction with Capsid rather than the Integrase protein [84]. Therefore, current experimental data indicates that TNPO3 is essential for PIC import, whereas the role for karyopherin-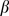 in this process remains unclear. Requirement for karyopherin-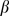 observed in [9] could be indirect, perhaps for transport of another HDF.

Budding, the release of the viral particle from the host cell plasma membrane, is an essential step in the HIV-1 life cycle. We identify two subsystems that have a role in budding: *protein localisation* and *ubiquitin*. Both of these groups include interactions involving HIV-1 p6, a region of the Gag protein that contains a late domain (L-domain). L-domains recruit host-cellular factors required by HIV-1 for budding. Our results indicate that p6 (along with other viral proteins) is ubiquitinated at the L-domain by three forms of ubiquitin (B, C and D). The p6 L-domain also interacts with subunits of the ESCRT-I complex, possibly via direct interaction with AIP-1/ALIX. These interactions, though not fully understood, have been shown to be important for HIV-1 budding [85] and are found in our results. These host factors are not identified among HDFs. However, HDFs include both ubiquitin-conjugating enzymes and ubiquitin-protein ligases. Therefore, it appears that ubiquitination plays an important role in HIV-1 replication that can be linked to viral budding.

In our results we define four subsystems where the host proteins and interactions contribute to HIV-1 provirus chromosomal integration and HIV-1 RNA transcription, namely, *regulation of transcription*, *DNA helicase*, *RNA polymerase activity* and *DNA integration*. The largest of these groups is *regulation of transcription* that includes many direct binding and co-stimulatory interactions between HIV-1 proteins Tat and Vpr, and host transcriptional regulators including: cyclin-dependent kinase 9 and cyclin T1 that form the Positive Transcription Elongation Factor b complex, general transcription factors TFIIF and TFIIH; NF
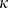
B; TATA box binding protein; cyclin-dependent kinase 9; CREB binding protein; p300; and p300/CBP-associated factor [86], [87]. Both the size of the intersection between this subsystem and HDFs and the proportion of genes annotated by GO as regulators of transcription is statistically greater than expected by random chance (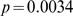 and 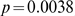, respectively). Transcriptional regulators we identify that are also among HDFs include cyclin t1, NF
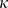
B, p300, TFIIF and TFIIH, as well as subunits of the RNA-polymerase II complex, as found in the *RNA polymerase activity* subsystem. Therefore, these host factors appear to form an essential functional module, with a clear pattern of interaction required for HIV-1 replication.

The *DNA integration* subsystem includes interactions between HIV-1 Integrase and three host proteins: LEDGF, a transcriptional activator; hSNF5, a subunit of the SWI/SNF ATP-dependent chromatin-remodeling complex; and embryonic ectoderm development (EED) protein. Integrase is involved in binding interactions with both LEDGF and hSNF5. LEDGF binds to Integrase and tethers it to host chromatin, an interaction identified as essential to HIV-1 infectivity [88], [89]. However, LEDGF is not found among HDFs, perhaps because this host factor is only required at a very low level, thus, eluding identification by siRNA knockdown screening [89]. This highlights the possibility that more host proteins shown to be essential for HIV-1 replication in specific, small-scale experiments may not be found among HDFs.

By cross referencing host proteins involved in significant patterns of interaction from the HHPID we have found support among siRNA screen data for host subsystems that can be linked to viral transcription, viral budding, PIC integration, transcription of viral RNA, changes to proteasome composition, export of viral RNA from the nucleus and regulation of apoptosis. However, of these, all but regulation of apoptosis and changes to proteasome composition might be considered an essential molecular mechanism for HIV-1 replication. Moreover, from our results it is unclear whether pro-apoptotic interactions are essential.

### Lack of support for T cell signaling and immune-related subsystems among HDFs

The remaining subsystems are not well supported by data from siRNA screens, in particular, those pertaining to cytokine dysregulation caused by HIV-1 infection. We do not find that HDFs are enriched for components of the TCR or for proteins involved in T cell activation. However, we do find that CD4 and CXCR4 have both been identified by our interaction patterns and by two siRNA screens, probably as these receptors were essential for virus entry in the two studies using HeLa cell lines [8], [10], whereas in the third study, CD4 and CXCR4 were not identified, presumably because an engineered mechanism for viral entry was employed [9].

There is little support for proteins involved in MAP kinase or PI3K-mediated intracellular signaling among global siRNA data sets. We find no HDFs that are GO annotated as having MAP kinase activity and just one HDF with lipid kinase activity (phosphatidylinositol-4-phosphate 5-kinase type-1-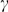), though we find two HDFs with PKC activity (PKC-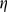 and serine/threonine-protein kinase N2). These findings indicate that knock-downs of single proteins from these cascades are generally insufficient to significantly inhibit HIV-1 replication. However, we surmise these central cascades are able to maintain signal transduction through multiple routes, with the KEGG representation of the MAP kinase cascade supporting this possibility [90]–[92]. Furthermore, HIV-1 interaction with these cascades is largely regulatory, rather than the result of direct interactions. Therefore, HIV-1 may not require any one specific protein from a central signal transduction cascade, such as a particular MAP kinase, for HIV-1 replication. Yet transduction of virally induced signals through the host cell is almost certainly an important mechanism in the proliferation of HIV-1.

We do not find that the subsystem annotations of any of *cytokine activity*, *interleukin*, *interferon*-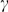 and *nuclear factor* subsystems represent statistically significant sets among HDFs. However, among HDFs, we do find five genes that are designated by GO as having cytokine activity including IL-1, chemokine-like factor, two additional interleukins (IL-18 and IL-22) and Interferon-related developmental regulator 2. These results indicate some cytokines and chemokines are likely to enhance HIV-1 replication. However, in our results cytokines form a far larger and more prominent set of host proteins and interactions. We suggest that this disparity is because while *in vivo* cytokines play a key part in modulation of viral replication, by providing a pool of cells for infection [93], immune system activation via cytokine release may not be essential for viral replication within any given cell. Indeed, *in vitro*, HIV-1 regulation of cytokines is likely to be of diminished importance as there is no functional acquired or innate immune system for the virus to interact with, either for the purpose of evasion or hyper-stimulation. Furthermore, small scale *in vitro* studies that have been explicitly designed to test the significance of HIV-1 protein interactions with cytokines and *in vitro* siRNA screens that test for HIV-1 dependence on host factors on a global scale, are unlikely to reach the same conclusion, regarding the relevance of cytokines to HIV-1 infection. The diminished importance of cytokines among HDFs is also indicated by the lack of support for the NFATs that promote cytokine transcription.

Innate cellular immune responses, such as APOBEC activity, the interferon system and TRIM22-induced interferon activation will be important *in vitro* [94]–[97]. Though as these innate immune factors exert a negative effect on HIV-1 replication, they are unlikely to be highlighted among HDF sets.

### Conclusion

By capturing the published knowledge of HIV-host interactions, the HHPID represents a hugely valuable resource for HIV-1 research. However, redundancy and heterogeneity of the PPI data, in terms of experimental methods, age of findings and quality of data, make the HHPID a difficult data set from which to draw conclusions about the overall system of HIV-1 infection, such as the identification of specific host functions and processes that are essential for HIV-1 replication. Using the strategy presented here, we identify significant patterns of HIV-host interaction, defined as sets of host proteins that take part in similar, enriched combinations of interactions during the course of HIV-1 infection. We have confirmed that these host protein sets, linked by their HIV-1 interaction profiles, are biologically related, tending to include proteins with common biological processes, proteins that share a high number of interactions with one another, subunits of the same complex and paralogs. In addition, we find that by identifying significant interaction patterns, we select for higher-confidence, well-studied interactions, based on the number of supporting journal articles. Hence, the identified higher-level groups, based on shared interactions, represent significant cellular subsystems used by HIV-1. Notably, our method incorporates the biological action of each PPI. Therefore, unlike other studies that identify cellular subsystems important to HIV-1 [1], [9], [11], the subsystems presented here, respect specific activity-related patterns of viral perturbation.

By assessing these subsystems using scientific literature and support from three global siRNA screen HDF sets, we have been able to describe systems of interaction that are invoked by HIV-1 to hijack host functions in order to successfully replicate including virus entry, mechanisms for viral gene transcription, export of viral RNA from the nucleus, viral budding and control of the proteasome. In addition, we also highlight mechanisms through which HIV-1 infection perturbs host processes at multiple cellular levels through a cycle of interactions that are not necessarily essential for viral replication, and appear detrimental to the human host by damaging the host immune response through dysregulation of cell surface receptor mediated signaling, signal transduction pathways, host gene expression, cytokine release and cell death.

Our approach permits a detailed study of the overlap between significant patterns of HIV-host interaction in the HHPID and HDFs. The modest overlap may be attributed to the fundamental difference in the methods of construction between the source data sets. The siRNA screens do not explicitly identify host cell proteins that undergo direct physical interactions with the proteins of HIV-1 or whose expression is altered during HIV-1 infection, as with many of the host cell proteins given in the HHPID. Rather, these screens are designed to identify host-cellular proteins that are required by the virus for replication. Therefore, HDF sets will not necessarily capture host proteins that are misregulated during HIV-1 infection, i.e., may perturb normal cellular responses, or host proteins that are potentially detrimental to HIV-1 infection, such as APOBEC3G [94]. In addition, each study has its own intrinsic bias. Particularly, the HHPID will be subject to study bias [98], where aspects of perceived medical importance, such as T cell depletion, receive greater attention. Whereas methods employed in each siRNA screen will be better tailored to picking certain host proteins over others. For example, one siRNA screen was specifically designed to discover host factors involved in the early stages of HIV-1 replication [9], while another used a viral strain that expresses a truncated Vpr protein and does not express Nef or Vpu [8]. In addition, the stage in the viral life cycle is also likely to be an important factor in determining the activation of PPI modules in the host cell [99], therefore, not all studies may capture the same results. Hence, the lack of overlap between these small and global-scale data sets is not unexpected.

The direct intersection between any one HDF set and the HHPID probably represents a small set of high-confidence HIV-1 interacting host proteins important to HIV-1 replication. However, analysis of this intersection alone is unlikely to provide a thorough insight into host defense mechanisms, perturbations caused by HIV-1 infection, or proteins that are essential to virus replication. We suggest that future experimental work could expand the core knowledge presented here. In particular, we suggest that proteins and pathways that are indicated by siRNA screen to be essential for HIV-1 replication, though otherwise poorly understood, are studied in greater detail to continue to bridge the knowledge gap between high and low throughput data sources. A successful example of this approach is conformation of TNPO3 as an essential protein for HIV-1 PIC import [83], [84] after initial identification as an HDF by [8], [9].

The HHPID data set has been used previously to validate HDF sets. Specifically HHPID interactions and host factors have been used in conjunction with HDFs to aid identification of well-connected subnetworks, corresponding to certain host cell functions prevalent among HDFs [9], [11]. Several of these subnetworks represent functions identified in our results including the proteasome and transcriptional regulation. However, we are not aware of any other work in which core host cell functions, represented in HHPID data, have been assessed in terms of their presence among HDFs.

In this study, we have used a computational approach to disentangle a complex set of interactions to provide an accessible map of core HIV-1-host interaction patterns for virologists. Our methodology can be generalised and take PPI data from any source. Hence, our work will contribute to defining core host subsystems for other pathogens, particularly as a reference against which results from increasingly prevalent high-throughput data sources might be compared. In addition, aiding prediction of currently undiscovered host-virus PPIs using interaction profiles may be possible. This could be done by taking the interaction profile of a given human protein, i.e., a ‘subject profile’, and comparing it with interaction profiles from other human proteins, i.e., a set of ‘query profiles’ to look for common interactions that are missing for the subject profile that are common to many other similar profiles (the distance measure in our work would be a method to quantify this commonality). However, this would form just part of such a prediction process and other established biological phenomena that impact upon PPI activity, such as interaction interfaces and cellular localisation, would also have to be considered to make successful predictions. Notably, our results and the potential predictive power to which we refer, are reliant upon an accessible and structured description of biological action for each PPI, as supplied in the HHPID. We have, thus, demonstrated that the inclusion of concise annotation in large-scale data can enhance resolution and allow greater depth of computational analysis.

## Materials and Methods

### Data collection

HIV-human PPI data were obtained from the HHPID on 1st May 2009. Specifically, distinct PPIs based upon: (i) the HIV-1 protein interactant, (ii) the human protein interactant and (iii) the type of interaction, one of 68 short descriptions that characterise the PPI outcome, were obtained [4], [5]. In cases where multiple transcripts of the same gene take part in the same interaction (with respect to HIV-1 protein and interaction type), only a single instance of the transcribed gene and interaction were used throughout our analyses.

To test whether interaction types are uniformly distributed among HIV-1 proteins, interaction types for each HIV-1 protein were counted and 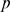-values were calculated using two-tailed Fisher's exact tests and corrected for multiple tests using the Benjamini and Hochberg [100] method.

### Bicluster identification

In order to perform biclustering, a binary matrix was created with one row per human protein and one column per HIV-1 interaction. We define an HIV-1 interaction to include both the HIV-1 interactant and the interaction type, e.g., ‘capsid activates’ is one such interaction. The presence of a given HIV-1 interaction, for a given protein, was represented in the matrix by a one and the absence by a zero (figure 2). To find sets of human proteins that share the same set of HIV-1 interactions in this matrix, biclustering was performed using the Bimax algorithm [101].

The significance of biclusters was determined by Monte Carlo simulation. Specifically, the HIV-human PPI network was rewired at random, while the degree of each protein and interaction type frequencies were maintained. The resulting network was used to produce a new matrix for biclustering. The matrix was biclustered using Bimax and interaction types, HIV-1 proteins and the number of human proteins in each bicluster were recorded. 50 000 iterations of this process were carried out. Using these simulations, we were able to empirically calculate the probability of randomly finding a bicluster involving a given number of human proteins and the same (or larger) set of HIV-1 interactions. Biclusters were deemed significant if they had a 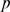-value of 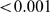, after correction for multiple tests using the Benjamini and Hochberg [100] method.

### Bicluster classification

All interaction types from the HHPID were organised into a hierarchy (see supplementary Text S1). This hierarchy included new parent terms. For example, a parent term ‘physical’ was created, the child terms of which all refer to more specific forms of physical interaction. In addition, every interaction was designated a *direction*, *polarity* and *control*. *Direction* refers to whether it is the HIV-1 protein acting upon the human protein or vice versa, e.g., ‘Tat inhibits p53’ has a forward direction, ‘Tat is inhibited by p53’ has a backward direction and ‘Tat interacts with p53’ has a neutral direction. *Polarity* refers to the biological action of the interaction, e.g., ‘Vpr activates p53’ has a positive polarity, ‘Vpr inhibits p53’ has a negative polarity and ‘Vpr interacts with p53’ has a neutral polarity. *Control* refers to regulation within the interaction, additional to the polarity, e.g., ‘Tat decreases phosphorylation of retinoblastoma 1’ has a positive polarity but due to the verb ‘decrease’ has a negative control, while ‘Tat increases phosphorylation of retinoblastoma 1’ has a positive control. For those interaction types with no additional control, we set control as null.

This information was used to classify biclusters according to the hierarchical relationship between their interactions. We defined three types of relationship between interactions: two positive relationships, parental and sibling, and one non-relationship, independence. Positive relationships refer to the same biological event within a given interaction, described using a different and perhaps more, or less specific term. Independence, denotes that two interactions describe distinct events, both providing additional information.

For any two interactions to be part of a positive relationship, they must link the same two protein interactants, their directions must not be opposing, i.e., forward and backward, their polarities must not be opposing and the control must be the same. Parental interaction relationships are formed when one interaction is the descendant of another, e.g., ‘Tat binds p53’ is a descendant of the interaction ‘Tat interacts with p53’. Sibling interaction relationships are formed when both interactions have the same direct parent term, e.g., ‘Tat activates Cdk2’ is a sibling of ‘Tat enhances Cdk2’, as the parent term for both interaction types is ‘protein regulation’. Interaction pairs that do not conform to parental or sibling relationships have an independent relationship. These relationship classifications give rise to five classes of bicluster: (i) *Independent*, where all interactions have independent relationships. (ii) *Parental*, where all of the interactions are descendants of one another. (iii) *Sibling*, where two or more interactions are siblings of one another in the ontology, e.g., ‘Tat activates Cdk2’ is a sibling of ‘Tat enhances Cdk2’ (iv) *Family*, where all of the interactions form a ‘family’ of parental and sibling relationships. (v) *Mixed*, where independent interactions and sibling or parental interactions form a bicluster.

### Bicluster biological validation

We established a group of 692 proteins from the HIV-1 interacting set that could appear in the bicluster results. These proteins are limited to those that have more than one distinct HIV-1 interaction. This set of proteins are important to our statistical analyses and will be referred to as potential bicluster proteins (PBPs).

A human PPI network was created using protein interactions derived from multiple sources: BioGRID [102], BIND [103] and HPRD [104]. All interactions were cross-referenced using the ‘gene_info’ file provided by the Entrez Gene database (ftp://ftp.ncbi.nlm.nih.gov/gene) to maintain consistent accession labeling. These data sets were obtained in July 2009. The human PPI network contained only one node per human gene, a maximum of one edge between two nodes and a total of 9000 nodes and 30478 edges.

The number of shared edges, average shortest path length and largest connected component (LCC) for the set of human protein nodes defined by each significant bicluster were calculated from this network and statistical significance was calculated by Monte Carlo simulation. In a single iteration of this simulation, a group of nodes numbering the same as the bicluster in question were selected at random using rejection sampling in order to maintain the group degree distribution. Following this, shared edge count, average shortest path length and LCC were recorded. 10000 iterations of this simulation were carried out per bicluster. The results of the simulation were used to estimate the probability that a more tightly clustered set of nodes, of given size and degree distribution would be found by random chance, 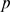-values were corrected for multiple tests using the Benjamini and Hochberg [100] method.

To analyze similarity between proteins within biclusters, we performed local protein sequence alignments between all PBPs using the Smith-Waterman algorithm [105] with a gap open cost of 10, a gap extension cost of 0.1 and the BLOSUM62 substitution matrix. To analyze similarity in annotation between proteins within biclusters, we carried out a semantic similarity measurement [106] between all PBPs using GO annotation for all three ontologies: molecular function, biological process and cellular component. The GO data was downloaded from the Gene Ontology on the 9th December 2008. We defined the distance between two genes using the method given in [26], using the semantic distance measurement defined in [106]. For both of these measures we compared the value distribution for protein pairs that appear in the same significant bicluster to the equivalent distribution for proteins that do not appear in the same significant bicluster using a Mann-Whitney U test. We also calculated 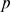-values for each significant bicluster, for both of these measures, using Monte Carlo simulations. For a given bicluster of size 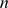 and a mean average alignment score, or semantic similarity, 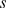, 100000 and 10000 simulation iterations, for the pairwise alignment and semantic similarity simulations, respectively, were performed. In each iteration we selected a non-redundant random set of 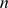 proteins from PBPs and calculated the average alignment score or semantic similarity and counted whether this value was greater than, or less than 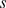. By this method we were able to calculate the probability of finding a set of proteins, by random chance, with greater similarity than the proteins of a bicluster, both in terms of sequence and GO annotation. We corrected the 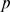-values for multiple tests using the Benjamini and Hochberg [100] method. In addition, we identified groups of similar human protein sequences within significant biclusters using single-linkage clustering; linking pairs of proteins that have 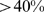 sequence identity, determined by sequence alignments and selecting connected components.

### Defining subsystems

The distance between any two biclusters, 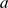 and 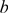, was calculated using the formula:
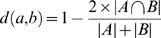



Where 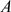 and 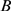 are the set of interactions in biclusters 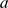 and 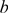, 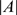 and 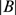 are the number of interactions in sets 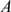 and 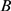 and 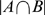 is the size of the intersection between sets 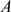 and 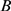. Therefore, for two identical biclusters 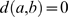, while for two biclusters that have no common interactions 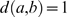. Distances between all biclusters were calculated, cubed to obtain a greater range of values and the resulting distances were used to define relationships between biclusters using neighbor joining [37]. Meaningful groups were determined, by examining bicluster proteins and interactions. These groups were characterised and named using one of the following two methods: (i) Selecting one or more over-represented GO term (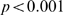), calculated using Fisher's exact tests corrected for multiple tests using the Benjamini and Hochberg [100] method. (ii) In the case where the proteins of a subgroup are all homologs or isoforms of the same product and no specific GO term pertaining to that protein product exists, a regular expression encapsulating the protein name was used to characterise the group and that group was named after the protein. The method used to define each group is specified in supplementary Table S3.

### Comparison with siRNA screen data

Proteins from three siRNA studies [8]–[10], were cross referenced against the identified host subsystems. These studies include 281 [8], 295 [9] and 290 [10] genes. The genes not expressed in T cells or macrophages, designated group ‘H’ in one study (reference [10]) were not included. The number of successful hits against each subsystem and the direct intersection was counted for each of the three studies and 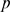-values for these counts were calculated using chi-square tests, using all genes annotated in the gene ontology as the population, corrected for multiple tests using the Benjamini and Hochberg [100] method. HIV-1-host PPI networks were constructed and visualised using Cytoscape [107].

## Supporting Information

Table S1 Table of significant biclusters and their HIV-host interactions. A table of significant biclusters. Each row represents a single HIV-host interaction within a significant bicluster. The biclusters are divided in to higher-level groups, known as sub-systems, based on shared interactions and labeled according to the biological role of the included host proteins. From right to left, the columns show: name of the sub-system; bicluster id; *p*-value for the bicluster; corrected *p*-value, calculated using the Benjamini and Hochberg FDR correction method; entrez gene id corresponding to the human protein interactant; name of the human protein interactant; entrez gene id corresponding to the HIV-1 protein interactant; a string identifier for the interaction type, consisting of a short HIV-1 protein name and a description of the interaction outcome, separated by an underscore; the relationship of that interaction to other interactions within the same bicluster; id for the corresponding interaction in the HHPID; the HHPID description of the interaction.
(2.80 MB XLS)

Table S2 Table of biological cohesiveness measures for significant biclusters. A table of biological cohesiveness measures. Each row represents a significant bicluster. Sequence similarity, semantic similarity and network clustering are measures pertaining to the proteins of a given bicluster. From right to left, the columns show: bicluster id; *p*-value for sequence similarity; corrected *p*-value for sequence similarity, calculated using the Benjamini and Hochberg FDR correction method; number of proteins found in the largest protein cluster, within that bicluster, determined by single linkage clustering, using a linkage cut-off of 40% sequence similarity; as in the latter but using a cut-off of 80% sequence similarity; *p*-value for human PPI network shared edge count; corrected *p*-value for human PPI network shared edge count, calculated using the Benjamini and Hochberg FDR correction method; *p*-value for human PPI network largest connected component; corrected *p*-value for human PPI network largest connected component, calculated using the Benjamini and Hochberg FDR correction method; *p*-value for human PPI network average shortest path length; corrected *p*-value for human PPI network average shortest path length, calculated using the Benjamini and Hochberg FDR correction method; *p*-value for semantic similarity using the GO biological process ontology; corrected *p*-value for semantic similarity using the GO biological process ontology, calculated using the Benjamini and Hochberg FDR correction method; *p*-value for semantic similarity using the GO cellular component ontology; corrected *p*-value for semantic similarity using the GO cellular component ontology, calculated using the Benjamini and Hochberg FDR correction method; *p*-value for semantic similarity using the GO molecular function ontology; corrected *p*-value for semantic similarity using the GO molecular function ontology, calculated using the Benjamini and Hochberg FDR correction method.
(0.09 MB XLS)

Table S3 Table of host subsystem details. A table of host subsystem details. Each row represents a host subsystem. From right to left the columns show: the name of the subsystem; the number of biclusters included in the subsystem; the number of human genes in the subsystem; the intersection between the subsystem and the *Brass et al.* (2008) siRNA screen; *p*-value for the latter; corrected *p*-value for the latter, calculated using the Benjamini and Hochberg FDR correction method; the intersection between the subsystem and the *Konig et al.* (2008) siRNA screen; *p*-value for the latter; corrected *p*-value for the latter, calculated using the Benjamini and Hochberg FDR correction method; the intersection between the subsystem and the *Zhou et al.* (2008) siRNA screen; *p*-value for the latter; corrected *p*-value for the latter, calculated using the Benjamini and Hochberg FDR correction method; the type of subsystem annotation used to identify the subsystem; the details of the subsystem annotation; the number of human genes from the sub-system that fit the subsystem annotation; the *p*-value for the subsystem annotation; a corrected *p*-value for the subsystem annotation, calculated using the Benjamini and Hochberg FDR correction method; number of genes from the *Brass et al.* (2008) siRNA screen that fit the subsystem annotation; *p*-value for the latter; corrected *p*-value for the latter, calculated using the Benjamini and Hochberg FDR correction method; number of genes from the *Konig et al.* (2008) siRNA screen that fit the subsystem annotation; *p*-value for the latter; corrected *p*-value for the latter, calculated using the Benjamini and Hochberg FDR correction method; number of genes from the *Zhou et al.* (2008) siRNA screen that fit the subsystem annotation; *p*-value for the latter; corrected *p*-value for the latter, calculated using the Benjamini and Hochberg FDR correction method.
(0.04 MB XLS)

Text S1 Hierarchy of protein interaction types. A hierarchy that incorporates all of the interaction types found in the NCBI HIV-1, host protein interaction database (HHPID) with the addition of parent terms for these types. HHPID interaction types have a unique id, and *polarity*, *direction* and *control* attributes. These attributes are explained in detail in the methods section in the main text of this article. Interaction types found in the HHPID are present as *instance* elements, parent terms are designated as *interactionType* elements.
(0.01 MB XML)
